# Profiling of UGT1A1^*^6, UGT1A1^*^60, UGT1A1^*^93, and UGT1A1^*^28 Polymorphisms in Indonesian Neonates With Hyperbilirubinemia Using Multiplex PCR Sequencing

**DOI:** 10.3389/fped.2019.00328

**Published:** 2019-08-07

**Authors:** Radhian Amandito, Rinawati Rohsiswatmo, Erica Carolina, Rizka Maulida, Windhi Kresnawati, Amarila Malik

**Affiliations:** ^1^Neonatal Intensive Care Unit, Pondok Indah General Hospital, Jakarta, Indonesia; ^2^Division of Perinatology, Department of Pediatrics, Faculty of Medicine, Cipto Mangunkusumo General Hospital, Universitas Indonesia, Jakarta, Indonesia; ^3^Division of Pharmaceutical Microbiology and Biotechnology, Faculty of Pharmacy, Universitas Indonesia, Depok, Indonesia; ^4^Department of Epidemiology, Faculty of Public Health, Universitas Indonesia, Depok, Indonesia; ^5^Neonatology Unit, Biak General Hospital, Biak Numfor, Indonesia

**Keywords:** Indonesia, snapshot, polymorphism, UGT1A1, unconjugated hyperbilirubinemia

## Abstract

**Background:** Single nucleotide polymorphism (SNP) variants of the uridine diphosphate glucuronosyltransferase 1A1 (*UGT1A1*) gene have been studied as an important factor in neonatal hyperbilirubinemia (jaundice) severity. Specific ethnicities, including Asians, have certain SNPs that appear more frequently than others.

**Aim:** To identify the most common SNPs in Indonesian neonates and their association with the severity of neonatal hyperbilirubinemia.

**Methods:** Eighty-eight inborn and outborn jaundiced infants from three different hospitals (Bengkulu, Jakarta, Biak Papua) across Indonesia were enrolled in this cross-sectional study and their peak total serum bilirubin (TSB) levels assessed. SNP variant analyses of the TATAA box, promoter, and exon 1 regions of UGT1A1 gene from 78 of the 88 infants were carried out using the SNaPshot^R^ Multiplex Polymerase Chain Reaction (PCR) System followed by DNA sequencing.

**Results:** We detected SNP variants UGT1A1^*^28, UGT1A1^*^60, UGT1A1^*^93, and UGT1A1^*^6 in our population. Mean total serum bilirubin (TSB) was 14.59 ± 5.57 mg/dL. Bivariate analyses using delivery location, gestational age, birth weight, mother's age, and ethnicity were shown to be associated with moderate-to-severe hyperbilirubinemia (*p* < 0.05). None of the four SNPs appeared to be associated with moderate-to-severe hyperbilirubinemia. In multivariate analysis, however, only the “other ethnic group” (e.g., Chinese, Bengkulu, Papua, Bima) category showed an association with moderate-to-severe hyperbilirubinemia, with an odds ratio of 6.49 (95% CI 1.01–41.67; *p* < 0.05).

**Conclusions:** We found that the UGT1A1^*^60 is the most common SNP detected in neonates with hyperbilirubinemia in the Indonesian population. Interestingly, in Indonesia, *UGT1A1* polymorphisms do not appear to be associated with differences in the severity of hyperbilirubinemia.

## Introduction

Neonatal hyperbilirubinemia (jaundice) is commonly-encountered in daily medical practice. Availability of early detection of hyperbilirubinemia enables clinicians to treat these patients promptly with phototherapy. This improvement in healthcare reduces the neonatal mortality and morbidity rates of jaundice-related conditions. In most cases, the neonatal jaundice is physiological. It is a benign transitional phenomenon related to immaturity and hemolysis of red blood cells, liver, and gastrointestinal system, in which the problem resolves with little to no therapy (1–3). However, in some cases, jaundice is pathological and with rapidly-increasing levels of TSB (total serum bilirubin) that requires urgent therapy and scrutinized observation (3). On studies have shown that the risk factors for hyperbilirubinemia consist of hemolytic diseases, as well as environmental and genetic origins, especially when multiple factors are co-expressed (1, 3, 4). The most common gene variants reported to be associated are those encoding for the enzyme G6PD (glucose-6-phosphate dehydrogenase), OATP/SLCO1B1 (hepatic solute carrier organic anion transporter 1B1), and UGT1A1 (uridine diphosphate glucuronosyltransferase 1A1) (1, 5). *UGT1A1* has been widely-studied in different populations and the findings appear to differ between ethnicities (6–9).

The human UGT1A1 is the enzyme responsible for bilirubin conjugation with glucuronic acid. *UGT1A1* genetic variants that lead to reduced activity and decreased expression of the enzyme have been shown to be associated with non-hemolytic hyperbilirubinemia syndromes such as Gilbert's syndrome (GS) and Crigler-Najjar's syndrome types I and II (CN I and CN II, respectively) (1, 10–15). A variant of the TATAA box that has an additional TA insertion (A(TA)7TAA) (UGT1A1^*^28) was found to be associated with GS and CNs, with the mutation being found most frequently in Caucasian and African populations (1, 13, 16–19). In the East Asian population, however, UGT1A1^*^6 (211G > A) is the most prevalent single nucleotide polymorphism (SNP) (16, 17, 20, 21). This variant leads to an amino acid substitution of glycine-71 to arginine. In the promoter region of the gene, variant UGT1A1^*^60 (3279T > G) was found to be a common SNP in Egyptian neonates with neonatal hyperbilirubinemia (22). UGT1A1^*^60 is a SNP located in the phenobarbital-responsive enhancer module (gtPBREM) associated with decreased transcriptional activity of the UGT1A1 promoter (11, 12, 18). More recently, UGT1A1^*^93 (3156G > A), a SNP located between the TATAA box and gtPBREM, was found to show a high extent of linkage disequilibrium between UGT1A1^*^93 and UGT1A1^*^28 (11).

We have previously found that the UGT1A1^*^6 and UGT1A1^*^60 SNPs were prevalent in the ethnic Bengkulu population in Indonesia (23). However, the prevalence of the UGT1A1^*^28 and UGT1A1^*^93 SNPs have not been previously investigated in Indonesia, with the only other studies performed with the Malaysian population (11, 13, 14). This study aimed to identify the most common SNPs associated with neonatal jaundice in the Indonesian population.

## Materials and Methods

### Study Population

This was a cross-sectional, multi-center study conducted in three hospitals across Indonesia: (i) M. Yunus General Hospital in Bengkulu province; (ii) Biak General Hospital in West Papua province; and (iii) Cipto Mangunkusumo Hospital in Jakarta. The data collection process lasted 12 months (September 2016 to September 2017). Eighty-eight neonates who were diagnosed with clinical jaundice (based on Kramer's index) (24). by a neonatologist between days 3–7 post-natal were enrolled. Both pre-term and full-term, in-born and out-born neonates were included. We applied exclusion criteria in order to obtain unbiased data to prove the extent of genetic factors. Exclusion criteria includes neonates with signs of hemolytic anemia including those caused by ABO incompatibility (assessed through blood grouping of mother and neonate, direct Coomb's test of neonate, and fall in Hemoglobin of neonate), cephalhematoma, sepsis, major congenital malformations, neonates with conjugated bilirubin >20% of the TSB, and G6PD deficiency. These conditions were evaluated through clinical and laboratory examinations. Out of the 88 neonates that were originally enrolled, several had very low levels of DNA present in the sample, thus complicating the SNP analysis. Peak TSB was measured from venous blood (taken between day 3 and 7 before phototherapy was administered) using the ADVIA Chemistry Total Bilirubin 2 device (25). Peak TSB levels <13 mg/dL indicated mild hyperbilirubinemia, and moderate-to-severe hyperbilirubinemia was indicated by levels ≥13 mg/dL (26). We used this classification based on Paludetto et al. (26) due to the slight variation period when the TSB was taken. Other variables obtained included gestational age, birth weight, sex, mother's age at delivery, delivery method, delivery location, exclusive breastfeeding, history of older sibling requiring phototherapy, and the parents' ethnicity.

The study was approved by the Health Research Ethics Committee—Universitas Indonesia and Cipto Mangunkusumo Hospital. We obtained prior consent from the parents of all included neonates in the study.

### Detection of SNPs

Peripheral blood samples were stored at −20°C in EDTA-containing vacutainer tubes until required. Genomic DNA (gDNA) was purified from blood samples using the QIAamp DNA Blood Mini Kit (QIAGEN, Germany) as described previously (23). The gDNAs were subsequently used for multiplex PCR experiments and SNP genotyping.

### Analysis of DNA Sample by PCR

All oligonucleotide primers used in this study are listed in Supplementary Table 1. Genotyping of three SNPs in the promoter and coding regions of *UGT1A1*, namely UGT1A1^*^60 (promoter, 3279T>G), UGT1A1^*^93 (promoter, 3156G>A), and UGT1A1^*^6 (Exon 1, 211G>A) was carried out using the SNaPshot^R^ Multiplex kit. Genotyping of the TATAA box sequence repeat (UGT1A1^*^28) in the UGT1A1 promoter region was determined by cloning and sequencing of PCR amplicons.

A total volume 25 μl of PCR Mix was prepared which consisted of 12.5 μl PCR Master Mix MyFi Mix^TM^ (Bioline, USA), 0.5 μl forward primer (10 μM), 0.5 μl reverse primer (10 μM), 1.0 μl DNA template (50 ng/μl), and 10.5 μl nuclease-free water. PCR was carried out using the following parameters: initial denaturation of 5 min at 95°C followed by 30 cycles of denaturation of 95°C for 30 s, annealing at 55°C for 30 s, extension at 72°C for 1 min, and a final polymerization at 72°C for 10 min. The presence of amplicons was confirmed by agarose gel electrophoresis (Supplementary Figure 1) prior to DNA sequencing. DNA sequence data were analyzed using the GeneMapper 4.0 (Applied Biosystems) system (1st BASE Sdn. Bhd., Malaysia).

Amplicons for cloning were amplified in 25 μl of PCR mix as follows; 12.5 μl PCR Master Mix (KOD FX Neo, Toyobo), 1.0 μl Forward primer (10 μM), 1.0 μl Reverse primer (10 μM), 1.0 μl DNA template (50 ng/μl), and 9.5 μl nuclease free water. PCR amplification was carried out as follows: initial denaturation of 3 min at 95°C followed by 35 cycles of denaturation of 95°C for 20 s, annealing at 55°C for 30 s, extension at 68°C for 30 min, and final extension at 68°C for 5 min. One microliter aliquots of individual unpurified 207-bp amplicons (Supplementary Figure 2) were then inserted into the pJET1.2 cloning vector (ThermoFisher, USA) and transformed into competent *Escherichia coli* DH5α cells according to the manufacturer's protocol (27). Colony PCR was used to screen for positive clones and recombinant plasmids purified from 18-h cultures were sequenced with both UGT1A1^*^28_F and UGT1A1^*^28_R primers (1st BASE, Malaysia).

### Statistical Analysis

STATA version 12 (Macintosh version) was used for data management and statistical analyses. The variables were first presented descriptively with 88 subjects. However, only 78 subjects were analyzed further for bivariate and multivariate analyses as 10 participants had to be excluded due to incomplete data. Bivariate analysis was conducted between independent and dependent variables using chi-square/Fisher's exact test, Student's *t*-test, and Kruskal-Wallis. Variables with *p* < 0.25 were included in the multivariate analysis using logistic regression. We used two-sided *p*-values in our analysis with a *p* < 0.05 level of significance. Gender, hospital, ethnic group, exclusive breastfeeding, delivery method, delivery location, sibling requiring phototherapy, ethnic groups, and SNPs were categorical data, while gestational age, birth weight, and mother's age were expressed as numerical data. Ethnicity was based on ethnic group majority.

## Results

Clinical characteristics of 88 neonates from all three study centers (Bengkulu, Jakarta, and Biak) are shown in Table 1. There was an almost equal proportion of female (54.5%) and male neonatal subjects (45.5%). More than half of the neonates were breastfed exclusively (60.2%), about half of the newborns were born via Cesarean-section (56.8%), and almost two-thirds were delivered in hospital (65.9%). Most of the neonates (96.6%) had 335 no sibling who required phototherapy. The neonates originated from many different ethnic groups with no dominant ethnic group. The average gestational age was 34.5 ± 3.7 weeks, average birth weight was 2189.1 ± 849.8 g, and average mother's age was 29.3 ± 6.2 years old. The mean peak TSB was 14.59 ± 5.57 mg/dL, and the number of neonates with severe hyperbilirubinemia (TSB > 25 mg/dL) is 4. Our bivariate analysis results of the clinical characteristic are shown in Table 2. Delivery location, gestational age, birth weight, mother's age, and ethnic groups are shown to be associated with moderate-to-severe hyperbilirubinemia (*p* < 0.05).

**Table 1:** Clinical characteristics of the neonates (*n* = 88).

**Characteristics**	***n* or Mean**	**% or SD**
Hospital		
Jakarta	42	47.7
Bengkulu	41	46.6
Papua	5	5.7
Gender		
Male	40	45.5
Female	48	54.6
Exclusive breastfeeding		
Yes	53	60.2
No	35	39.8
Delivery method		
Vaginal	38	43.2
Cesarean	50	56.8
Delivery location		
Midwife	30	34.1
Hospital	58	65.9
Sibling requiring phototherapy (*n* = 87)		
Yes	3	3.5
No	84	96.6
Ethnic group		
Javanese	12	13.6
Betawi	9	10.2
Sundanese	11	12.5
Minangkabau	5	5.7
Others	51	57.9
Gestational age, week	34.47	3.7
Birth weight, g	2189.15	849.8
Mother's age, year (*n* = 86)	29.28	6.2
Peak TSB, mg/dL	14.59	5.57
Peak tsb categorized, mg/dL		
Mild	35	39.8
Mild (mean, SD)	9.2	2.4
Moderate-to-severe	53	60.2
Moderate-to-severe (mean, SD)	18.1	4.0

**Table 2:** Variables associated with the severity of hyperbilirubinemia.

	**Hyperbilirubinemia**			
**Factors**	**Moderate-to-severe**	**Mild**	***P-*value**	**Crude prevalence odds ratio (CPOR)**
Gender				
Female	28 (56%)	14 (50%)	0.61^*^	1.27 (0.45–3.56)
Male	22 (44%)	14 (50%)		
Exclusive breastfeeding				
Yes	29 (58%)	19 (67.86%)	0.391^*^	0.65 (0.22–1.90)
No	21 (42%)	9 (32.14%)		
Delivery method				
Vaginal	25 (50%)	9 (32.14%)	0.127^*^	2.11 (0.73–6.34)
Cesarean	25 (50%)	19 (67.86%)		
Delivery location				
Midwife	20 (40%)	3 (10.17%)	0.009^**^	**5.56 (1.38–31.91)**
Hospital	30 (60%)	25 (89.29%)		
Sibling requiring phototherapy				
Yes	2 (4%)	0 (0%)	0.534^**^	N/A
No	48 (96%)	28 (100%)		
Gestational age				
(Mean ± SD, wk)	32.96 ± 3.39	35.28 ± 3.44	0.0054^****^	N/A
Birth weight				
(Mean ± SD, g)	1818.39 ± 745.072	2405± 838.81	0.0029^****^	N/A
Mother's age				
(Mean ± SD, y)	31.21 ± 7.28	28.06 ± 5.51	0.0342^****^	N/A
Ethnic group				
Javanese	5 (17.86%)	6 (12%)	<0.001^***^	N/A
Betawi	6 (21.43%)	3 (6%)		
Sundanese	9 (32.14%)	2 (4%)		
Minangkabau	4 (14.29%)	1 (2%)		
Others	4 (14.29%)	38 (76%)		

*Significant results are indicated in bold text*.

* *chi-squared*.

** *chi-squared with Fisher's exact test*.

*** *Kruskal-Wallis*.

**** *Student's t-test*.

The peak TSB measurements in neonates in the wild-type group and all UGT1A1 SNP variants are shown in Table 3. UGT1A1^*^6 only yielded 84 results with most neonates were wild-type (90.48%), UGT1A1^*^60 yielded 86 results with half of the neonates being wild-type (50%), and UGT1A1^*^93 only yielded 82 results with majority of neonates being wild-type (80.49%). In UGT1A1^*^28, almost all neonates were wild-type (97.73%). Bivariate analysis results of SNPs and hyperbilirubinemia are shown in Table 4. None of the SNPs showed any association with moderate-to-severe hyperbilirubinemia.

**Table 3:** Frequency of SNPs detected.

**Mutations**	***N***	**%**
UGT1A1*6 (*n* = 84)		
G/G (wild-type)	76	90.5
G/A (heterozygote)	8	9.5
UGT1A1*60 (*n* = 86)		
T/T (wild-type)	43	50.0
T/G (heterozygote)	34	39.5
G/G (homozygote)	9	10.5
UGT1A1*28 (*n* = 88)		
A(TA)6TAA	86	97.7
A(TA)6TAT (mutant)	1	1.1
A(TA)7TAA (mutant)	1	1.1
UGT1A1*93 (*n* = 82)		
G/G (wild-type)	66	80.5
G/A (heterozygote)	15	18.3
A/A (homozygote)	1	1.2

**Table 4:** SNP distribution in mild and moderate-severe hyperbilirubinemia groups.

**Mutation**	**Hyperbilirubinemia**			
	**Moderate-to-severe**	**Mild**	***P*-value**	**Crude prevalence odds ratio (CPOR)**
UGT1A1*6				
G/A (heterozygote)	4 (8%)	3 (10.71%)	0.697^**^	0.72 (0.11–5.36)
G/G (wildtype)	46 (92%)	25 (89.29%)		
UGT1A1*60				
T/G (heterozygote)	21 (42%)	12 (42.86%)	0.652^***^	1.05 (0.36–3.05)
G/G (homozygote)	4 (8.00%)	1 (3.57%)		2.4 (0.21–125.93)
T/T (wildtype)	25 (50%)	15 (53.57%)		Ref
UGT1A1*28				
A(TA)6TAT (mutant)	1 (2%)	0 (0%)	N/A^*^	N/A
A(TA)6TAA	49 (98%)	28 (100%)		
UGT1A1*93				
G/A (heterozygote)	9 (18%)	5 (17.86%)	0.987^*^	1.01 (0.26–4.31)
G/G (wildtype)	41 (82%)	23 (82.14%)		

* *chi-squared*.

** *chi-squared with Fisher's exact test*.

*** *Kruskal-Wallis*.

As shown in Table 5, variables with *p* > 0.25 from bivariate analyses (Tables 2, 4) were put into multivariate analysis using logistic regression. Only the minority, i.e. others (Chinese, Bengkulu, Papua, Bima) ethnic group showed association with moderate-to-severe bilirubinemia (*p* = 0.049) with a POR of 6.49 compared to Javanese neonates.

**Table 5:** Variables used in logistic regression analysis.

**Factor in study**	**OR (95% CI)**	***P***
Delivery method		
Vaginal	Ref	0.209
Cesarean	0.37 (0.07–1.77)	
Delivery location		
Hospital	Ref	0.576
Midwife	1.81 (0.23–14.39)	
Gestational age	0.85 (0.57–1.26)	0.424
Mother's age	0.94 (0.85–1.06)	0.316
Birth weight	1.00 (0.99–1.00)	0.063
Ethnic group		
Javanese	Ref	
Betawi	0.43 (0.05–3.86)	0.456
Sundanese	0.17 (0.02–1.67)	0.127
Minangkabau	0.35 (0.02–6.15)	0.472
Others^*^	**6.49 (1.01–41.67)**	**0.049**

*Significant results are indicated in bold text*.

* *Chinese, Bengkulu, Papua, Bima*.

UGT1A1^*^60 (promoter, 3279T > G), UGT1A1^*^93 (promoter, 3156G > A), and UGT1A1^*^6 (Exon 1, 211G > A), as well as TATAA box sequence repeat of promoter, UGT1A1^*^28, were considered the most frequently reported SNPs as previously studied in several Asian countries (5, 10, 28). We also previously studied UGT1A1^*^60 and UGT1A1^*^6 in Bengkulu, Sumatra population performing PCR-RFLP (23). Other three SNPs, i.e., c.686C4A, c.1091C4T, and c.1456T4G, were not detected.

## Discussion

The present investigation has confirmed and expanded on our previous findings on the association of UGT1A1^*^60 and UGT1A1^*^6 with neonatal hyperbilirubinemia in Indonesia (23). In addition, the present SNP analysis has also included two further SNPs, UGT1A1^*^28, and UGT1A1^*^93 to determine their prevalence, if any, in the condition. Furthermore, we have increased the ethnic diversity of patients to ensure a more accurate representation of Indonesia's multi-ethnic population.

Certain characteristics of our population, e.g., preterm mean gestational age, low mean birth weight, and more exclusive breastfeeding, were as expected (29). Previous studies reported that premature birth is associated with a defective UGT1A1 protein causing immaturity of the conjugating enzyme, therefore increasing the risk of severe hyperbilirubinemia (30, 31). In addition, prematurity is also associated with higher red blood cell, hepatocyte, and gastrointestinal cell immaturity leading to hemolysis and, consequently, a build-up of bilirubin (24, 32). Exclusive breastfeeding is also a very well-known risk factor for hyperbilirubinemia in the form of breastmilk jaundice (2, 10). Breastmilk jaundice may occur due to certain factors present in human breastmilk (i.e., IL 1ß, IL6, ß-glucuronidase, epidermal growth factor, alpha-fetoprotein), which inhibits the conjugation of bilirubin that facilitates its excretion (33). Chou et al. (10) reported an association between *UGT1A1* polymorphisms and breastfeeding jaundice (10). They concluded that both factors have to be present in order for higher development of hyperbilirubinemia to occur. This might be why not all neonates harboring the UGT1A1^*^28 polymorphism and with exclusive breastfeeding suffer from hyperbilirubinemia, and *vice versa*. Our current findings did not support this hypothesis. In addition, our term and healthy infants start breastfeeding in the delivery room when considered clinically stable. The pathophysiology of breastmilk jaundice involves the occurrence of weight loss after birth subsequent to fasting (34). Through immediate breastfeeding, we suspect that the expected increase in TSB due to breastmilk jaundice does not occur in our population. This might explain why breastfeeding is not significant in our study. Bivariate analysis also revealed that the delivery location [OR 5.56 (1.38–31.91)] and the mother's age were significant. Infants born in a hospital are more routinely examined for comorbidities including hyperbilirubinemia during the early days post-natal when they are still in hospital. It is therefore safer for mothers who have known risk factors, or history of a child with severe hyperbilirubinemia, to deliver their child in a hospital as they can identified early and treated appropriately. In North America, hyperbilirubinemia is the most important reason for readmission of neonates to the hospital (35). With regard to the mother's age, this has always been considered as a risk factor and predictive of extreme neonatal hyperbilirubinemia (36). In contrary, our study showed that infants of an older mother appears to be more protected against moderate-to-severe hyperbilirubinemia. We have to consider that the maternal health and antenatal examinations are inadequate in terms of quality in Indonesia (37). There may also be more younger mothers who do not attend routine antenatal visits due to lack of knowledge (38). In the Indonesian population, it is likely that a combination of different risk factors eventually leads to a higher risk of moderate-to-severe neonatal hyperbilirubinemia.

In previous *in vitro* studies, the UGT1A1^*^6 mutation has been proven to have decreased isozyme activity (5). Heterozygous forms display 60% lower activity, while the activity in homozygous forms is even less at <35%. Whereas, the presence of UGT1A1^*^6 is known to be a risk factor for mild adult hyperbilirubinemia (GS) in Caucasian and East Asian populations (16, 19, 28, 39), the prevalence of this SNP is low in the Southeast Asian population (17, 23, 28). Our study confirms this with higher occurrence of wild-type *UGT1A1* than the UGT1A1^*^6 SNP (90.5 vs. 9.5%) with an OR of 0.72 (0.11–5.36). When comparing between the mild and moderate-to-severe hyperbilirubinemia groups, we also did not find a significant association (*p* = 0.69). This shows that UGT1A1^*^6 is not prevalent, and that it is not a contributing factor toward the severity of hyperbilirubinemia in Southeast Asian populations.

We found a similar frequency of UGT1A1^*^60 SNPs in our population compared to wild-type. Those with this SNP are widely-known to have decreased transcriptional activity of UGT1A1 (about 60%) via an impaired gtPBREM (12, 18). This finding, however, contrasts what was reported for the Malaysian population, in which a higher frequency of UGT1A1^*^60 SNPs was observed (11). Interestingly, whilst the peak TSB measurements were higher in wild-type compared to the ^*^60 variants, the differences were not statistically significant (*p* = 0.652). SNPs of ^*^60 was found to co-exist with ^*^28 (40). It has been hypothesized that co-existence of both ^*^60 and ^*^28 SNPs would decrease the transcriptional activity of UGT1A1 by 70% (18). However, our study shows that no association could be found between individual nor multiple SNPs with developing moderate-to-severe hyperbilirubinemia compared to mild hyperbilirubinemia [OR of 1.05 (0.36–3.05) for heterozygotes and 2.4 (0.21–125.93) for homozygotes].

Another commonly found SNP combination that has been reported is UGT1A1^*^93 and UGT1A1^*^60. The ^*^93 SNP can be found not only in hyperbilirubinemic neonates, but also healthy neonates (4). The co-existence with ^*^60 SNPs can also explain this marked increase in TSB level. In our study, we found only a few number of ^*^93 SNPs and their existence was not predictive of moderate-to-severe hyperbilirubinemia in Indonesian neonates with an OR of 1.01 (0.26–4.31) (*p* = 0.98). This is consistent with findings from a Chinese study and a study comparing Uzbekistan and Japanese populations, in which wild-type ^*^93 were more frequent than their SNPs (41, 42).

In previous studies, the enzyme activity in UGT1A1 with TA insertions in the normal sequence A(TA)_6_TAA of the TATAA box promoter region significantly reduces enzyme activity (60–80% of normal) (2, 11, 39). However, in another functional study, longer TA repeats appear to be a protective factor for hyperbilirubinemia. According to the American Academy of Pediatrics, whereas Caucasian and Asian populations are more susceptible to hyperbilirubinemia due to the presence of longer TA repeats, African populations are less susceptible (43).

On the other hand, fewer TA repeats, which causes an overall increase in UGT1A1 expression, has been reported to reduce TSB levels in African neonates (4, 44). However, data on Southeast Asian populations remain inconclusive. For example, Yusoff et al. (14) showed a low incidence of this SNP, i.e., no significant association, with severe hyperbilirubinemia in Malaysian neonates (14). This is supported by our data for the Indonesian population which showed only one sample exhibiting this SNP (having moderate-to-severe hyperbilirubinemia). Considering our relatively low sample size, this result should be addressed, not only with a much larger population size, but also a more ethnically-diverse one.

UGT1A1^*^28 and UGT1A1^*^60 have been demonstrated to show strong linkage disequilibrium (40), and co-expression in these two promoter regions decreases the transcriptional activity to 37% of normal, more so when the homozygous alleles are present (30% of normal) (18). In our study, very few ^*^28 SNPs were found and therefore we could not prove this effect of this SNP combination. We should always keep in mind however, that the clinical manifestation of hyperbilirubinemia, especially moderate-to-severe hyperbilirubinemia, would not be present unless active hemolysis is occurring. Therefore, it is of vital importance that assessment of the causes of hyperbilirubinemia in infants also always includes factors related with hemolysis.

From the bivariate analysis, we included several variables that are eligible for multivariate analysis. We found that the only significant factor is ethnicity, specifically the groups comprising other ethnicities (Chinese, Bengkulu, Papua, and Bima; *p* = 0.049). Due to the small number of samples from individual ethnic groups, we were unable to identify which ethnic group has a higher risk factor toward moderate-to-severe hyperbilirubinemia in Indonesia. What we can infer from this is that there are intra-ethnic differences of *UGT1A1* polymorphisms in the Indonesian population, not unlike that observed in the Han, She, and Dong populations in China (45). However, we found that there is no significant association between ethnicity and all of the SNPs that we studied (*p* > 0.05). This prompts further investigation on what could differentiate between the ethnicities, both from their clinical and genetic factors that could be involved in the development of hyperbilirubinemia.

UGT1A1 is not only involved in neonatal hyperbilirubinemia, but certain variants are also important in drug delivery. One of them, UGT1A1^*^60, is located in the gtPBREM. The assessment of this genetic variation may be useful to consider phenobarbital treatment depending on their respective ethnic group populations. Considering the varied genetic makeup of Indonesia and Southeast Asian populations, even compared to Indian and East Asian populations, further studies in not only UGT1A1, but other genes, e.g., HMOX1, BLVRA, and SLCO1B1, especially those closely-related with hyperbilirubinemia or drug delivery are warranted (46–48). Approach and treatment of infants with hyperbilirubinemia and other *UGT1A1-*related drug therapies should be based on the considerations of SNPs specific for Southeast Asian populations.

There were several limitations to the present study. Firstly, bilirubin measurement timing was not possible to be taken at all exactly the same time post-natal. However, we ensured that the TSB level that we analyzed was at its peak for each neonate. Secondly, the results of this study could not be generalized to all neonates in Indonesia due to the limited number of cases and very limited number of participating hospitals/provinces. Additionally, discrepancy between numbers of samples that were available for analysis in different groups might also cause some experimental bias. The study design that we prefer is case control study, but due to limitations in the execution of our research we only were able to conduct a cross-sectional study. We also excluded most of the risk factors of hyperbilirubinemia, however this becomes a limitation since many other studies concluded that other risk factors aside from genetic polymorphism need to be present to result in a severe hyperbilirubinemia. A more comprehensive future investigation with a much larger sample size from other regions of Indonesia, is needed to define a nationwide correlation of genomic variation and hyperbilirubinemia severity in Indonesia.

In conclusion, our current study found that UGT1A1^*^6, UGT1A1^*^60, UGT1A1^*^93, and UGT1A1^*^28 are present in Indonesian population. The distribution and frequency of SNPs in our Indonesian population is, however, different when compared to other Asian populations, as only UGT1A1^*^60 was found to be prevalent, i.e., in at least half of the neonates we studied. No SNP in our study was found to be associated with the severity of hyperbilirubinemia in Indonesian neonates. Out of all the clinical factors, only ethnic grouping was considered significant and therefore future studies of other genes relevant to hyperbilirubinemia in Indonesia should include a more ethnically-diverse population.

## Data Availability

The raw data supporting the conclusions of this manuscript will be made available by the authors, without undue reservation, to any qualified researcher.

## Author Contributions

This work was carried out in collaboration between all authors. RA, RR, and AM designed the study. RA and WK acquired the data. RA, EC, and AM performed experiments. RA, RM, EC, RR, and WK, analyzed the data. RA, RM, and AM wrote the manuscript. EC, WK, and RR gave technical support and conceptual advice. All authors read and approved the final manuscript.

### Conflict of Interest Statement

The authors declare that the research was conducted in the absence of any commercial or financial relationships that could be construed as a potential conflict of interest.

## Abbreviations

CN: Crigler-Najjar Syndrome
G6PD: glucose-6-phosphate dehydrogenase enzyme
gDNA: Genomic DNA
GS: Gilbert's syndrome
gtPBREM: phenobarbital-responsive enhancer module
OATP/SLCO1B1: hepatic solute carrier organic anion transporter 1B1
PCR-RFLP: Polymerase Chain Reaction-Restriction Fragment Length Polymorphism
POR: prevalence odds ratio
TSB: total serum bilirubin
UGT1A1: UDP-glucoronosyltransferase 1A1.
