# Supplementary figures and images for: Profiling of UGT1A1*6, UGT1A1*60, UGT1A1*93, and UGT1A1*28 Polymorphisms in Indonesian Neonates With Hyperbilirubinemia Using Multiplex PCR Sequencing
Source: Front Pediatr. 2019 Aug 7;7:328. doi: 10.3389/fped.2019.00328 (PMC6693044; doi:10.3389/fped.2019.00328)

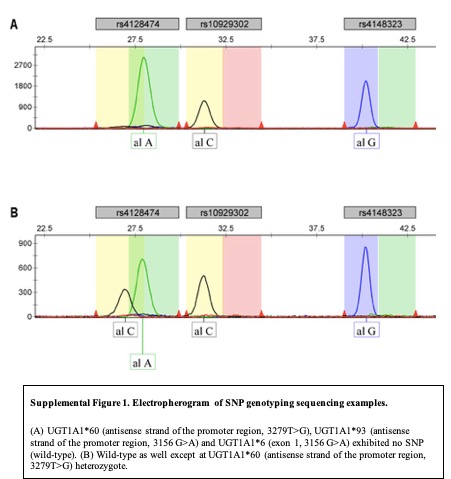

Supplement: Supplementary file 1 [file Image_1.JPEG]

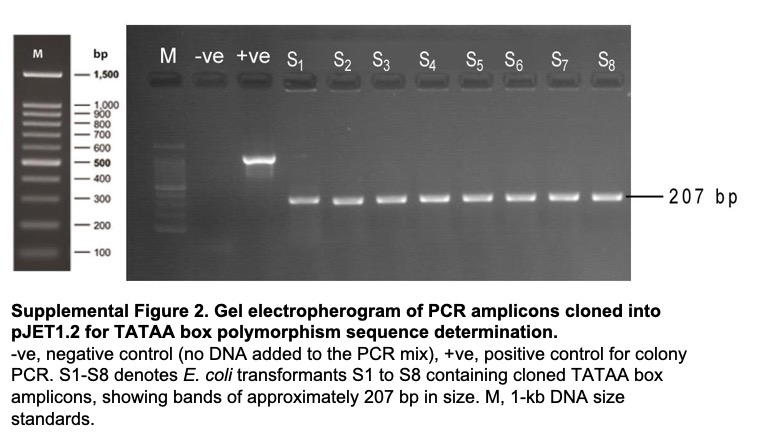

Supplement: Supplementary file 2 [file Image_2.JPEG]
