# Supplementary material for: Profiling of UGT1A1*6, UGT1A1*60, UGT1A1*93, and UGT1A1*28 Polymorphisms in Indonesian Neonates With Hyperbilirubinemia Using Multiplex PCR Sequencing
Source: Front Pediatr. 2019 Aug 7;7:328. doi: 10.3389/fped.2019.00328 (PMC6693044; doi:10.3389/fped.2019.00328)
Supplement: Supplementary file 3 [file Table_1.DOCX]

**Supplementary Table 1. List of oligonucleotides used in this study**

| No | Primer name and description | Sequence (5’-3’) | Oligonucleotide length (nt) | Expected PCR product size (bp) |
| --- | --- | --- | --- | --- |
| **Multiplex PCR** | | | |  |
| 1 | UGT1A1*93_UGT1A1*60_F  (multiplex forward primer) | AGTTCTCTTCACCTCCTCCTTA TTC | 25 | 561 |
| 2 | UGT1A1*93_UGT1A1*60_R  (multiplex reverse primer) | CCTTCTGAATCATTGCATCGGC TG | 24 |  |
| 3 | Probe UGT1A1*60 (detect T/G SNP) | GCCAAGGGTAGAGTTCAGT | 19 | 21 |
| 4 | Probe UGT1A1*93 (detect A/G SNP) | CTAGAGAGGAGGAATGAGCTT | 21 | 31 |
| 5 | UGT1A1*6_F  (multiplex forward primer) | TAACTTGTTCACTACATAGTCG TCC | 25 | 752 |
| 6 | UGT1A1*6_R  (multiplex reverse primer) | CTTGTTGTGCAGTAAGTGGGA AC | 23 |  |
| 7 | Probe UGT1A1*6 (detect A/G SNP) | GCCTCGTTGTACATCAGAGAC | 21 | 41 |
| **PCR Cloning** | | | |  |
| 5 | UGT1A1*28_F  (forward primer for cloning) | AAG TGA ACT CCC TGC TAC C | 19 | 207 |
| 6 | UGT1A1*28_R  (reverse primer for cloning) | CAG CAC ACA CAG CAG CA | 17 |  |
